# Stability of Synthesized Brushite in Physiological Media for the Possible Bone-Regenerative Use

**DOI:** 10.1155/ijbm/9636002

**Published:** 2025-07-02

**Authors:** Vilma Delgado-Morales, Lizbeth Barragán-Maldonado, Mercedes Salazar-Hernández, Alfonso Talavera-Lopez, Alba N. A. Ardila, Oscar Joaquín Solis-Marcial, Jose A. Hernández

**Affiliations:** ^1^Del Instituto Politécnico Nacional, UPIIG, Guanajuato, Mexico; ^2^Departamento de Ingeniería en Minas, Metalurgia y Geología, División de Ingenierías, Universidad de Guanajuato, Guanajuato, Mexico; ^3^Unidad de Ciencias Químicas, Universidad de Zacatecas, Campus UAZ Siglo XXI, Zacatecas, Mexico; ^4^Politécnico Colombiano Jaime Isaza Cadavid, Medellín, Colombia; ^5^Instituto Politécnico Nacional, UPIIZ, Zacatecas, Mexico

**Keywords:** brushite, hydrohyapatic, monetite, SBF, SIF, transformation

## Abstract

The advancement of science and technology has helped humans solve different problems related to their health. Among these applications are biomaterials, which are materials synthesized by humans for medical or biological use, representing a market and innovation with potential. The best known biomaterials are calcium phosphate cements (CPCs) that are used as bone substitutes, which show a similarity to bone minerals such as apatites such as dicalcium phosphate dihydrate, and it was synthesized and tested in previously prepared simulated intestinal and body fluids to analyze its stability under specific physiological conditions. Purity was determined by the ash method, giving an average of 73% and 85% in the different tests carried out. The characterization study was involved using ATR-FTIR, XRD, SEM, and EDS where changes were observed in the crystalline structure, in the bonds of the functional groups present on the surface and the morphology of Brushite causing the interaction with the different simulated fluids transformation into monetite, amorphous dicalcium phosphate, and hydroxyapatite.

## 1. Introduction

Research into new biomaterials as bone substitutes is a field of great importance in medicine and biotechnology to improve the regeneration and repair of bone tissue in the human body [1–5]. There are bioabsorbable synthetic polymers that have the ability to degrade due to hydrolysis reactions [6–10]. The choice of the appropriate biomaterial depends on the specific application and factors such as biocompatibility, mechanical strength, and chemical properties required for the desired function [11–14]. The mineral apatite is a natural calcium phosphate, the most abundant on the Earth's surface, and is the main source of phosphorus, making it of great importance in different fields of the biomaterial industry [15–20]. Calcium phosphate cements are compounds used clinically as bone substitutes, which constitute the most important mineral phase of the hard tissues of vertebrates [8, 21]. Various studies have shown the similarity between bone mineral and calcium phosphate minerals with an apatite structure [8, 10, 16, 22]. This makes calcium phosphates of natural or synthetic origin one of the most used routes in bone or dental surgery, when a supply of material is necessary [23, 24]. Among calcium phosphates, one of the most widely used minerals for orthopedic use is theoretical brushite (dicalcium phosphate dihydrate [DCPD]), commonly found in phosphate deposits and can be a component of sedimentary rocks, bird guano, and thermal water tanks [25–29]. DCPD can be obtained by different techniques, from expensive ones, such as plasma-assisted thermal projection, to low-cost techniques, such as chemical and electrochemical ones [25–30]. DCPD cements are biocompatible despite their acidic pH, which they produce in their environment after implantation, without producing any type of tissue reaction in response to the change in pH [1, 31, 32]. This effect is probably due to the buffer effect of the in vivo medium; it presents a higher reabsorption rate in animals implanted with this material [1, 31, 32]. Among the relevant characteristics is the solubility in water, allowing the controlled release of calcium and phosphate ions, which are essential for the mineralization of bone tissue [8, 17, 28]. Another characteristic of DCPD is its ability to adsorb biological molecules, making it an ideal scaffold for cell growth and the formation of new bone tissue [8, 10, 16, 17, 28]. After a period of implantation, biomaterials give rise to products that are not toxic and can be eliminated by the body or metabolized because the particles produced by wear cause an aggressive foreign body reaction that causes damage to the bone tissue [2, 3, 7, 27]. For this reason, the search for biomaterials with greater compatibility with the human body is of utmost importance because it remains a problem to be solved today [33–35]. The Ca/P molar ratio is important in the context of biomaterials because in biomedical and tissue engineering applications [1–3, 5, 10], maintaining an appropriate Ca/P ratio for biomaterial design is crucial for ensuring compatible with biological tissues and can interact effectively with them [10, 14, 36]. Furthermore, this ratio is also used as an indicator of the quality and composition of the materials to know what type of apatite it is: theoretical hydroxyapatite (HAP), DCPD, and anhydrous dicalcium phosphate (DCPA) [1–3, 5, 10, 14, 36]. The use of simulated body fluid (SBF) in biomaterials for bone substitutes has become a simple, inexpensive method that serves as an indicator of the bioactivity of biomaterials [4, 9, 18, 20]. The interaction of SBF with apatite is capable of forming apatite on its surface; when immersed in SBF, it can create direct bonds with the bone through this layer of apatite that forms on its surface in vivo, as long as the material does not contain any substance that induces toxicity or antibody reactions [4, 9, 18, 20, 26, 37]. Simulated intestinal fluid (SIF) is used to study the dissolution and precipitation of DCPD in the gastrointestinal tract since it is especially relevant to understand the mechanism of CaP formation [38–40]. In a complex fluid as SIF, simulates in vivo conditions due to the presence of amorphous calcium-magnesium phosphate (AMCP) nanoparticles [38–42]. Based on this, DCPD was evaluated under conditions similar to the biological environment to assess its efficacy in bone regeneration applications. DCPD was immersed in body and intestinal fluid to study the biomaterial's behavior and the release of calcium and phosphate ions in a simulated physiological environment.

## 2. Methodology

### 2.1. Brushite Synthesis (BCP) by Coprecipitation Method

The synthesis of brushite (BCP) was carried out by the coprecipitation method preparing to 0.033 M of Na_2_HPO_4_ ∗ 7 H_2_O (Jalmek, 100%) and 0.033 M of NaH_2_PO_4_ ∗ H_2_O (Jalmek, 100%) solution in 150 mL of deionized water, and the pH was adjusted to five with glacial acetic acid (CH_3_COOH, J. T. Baker, 99.7%) at a stirring speed of 120 rpm. On the other hand, to 50 mL, 0.2 M calcium acetate solution was prepared (Ca(CH_3_COO)_2_ ∗ H_2_O (Jalmek, 100%) which was added at a rate of 2 mL/min to the stirring phosphate solution. After the addition, the solution was stirred for another 10 min, filtered, washed with distilled water, and dried overnight at 37°C [27, 28].

### 2.2. Preparation of SBF

For 1 L of solution, 8.350 g of NaCl (Meyer, 100%), 0.355 g of NaHCO_3_ (Jalmek, 100%), 0.55 g of KCl (J. T. Baker, 99%), 0.231 g of K_2_HPO_4_ (Jalmek, 100%), 0.311 g of MgCl_2_ (Fermont, 100%), 0.292 g of CaCl_2_ (Karal, 100%), 0.072 g of Na_2_SO_4_ (Fermont, 100%), and 6.118 g of Tris (Karal, 100%) were weighed and finally 39 mL of HCl (J. T. Baker, 37%) were added to the solution. All components were then added to a 1-L flask and topped up with deionized water. Once topped up, the mixture was emptied into an amber bottle, a gauze stopper was placed, and the mixture was sterilized in an autoclave (Felisa) at 121°C, 15 lb_2_/in^2^ for 15 min [43].

### 2.3. Preparation of SIF

About 6.8 g of KH_2_PO_4_ (Jalmek, 100%), 2 g of NaOH (Jalmek, 100%), and 2 g of pancreatin (Sigma-Aldrich, CAS: 8049-47-6) were weighed, and they were filled into a 1-L flask with deionized water. Subsequently, it was emptied into an amber bottle, and a gauze stopper was placed and sterilized in the autoclave (Felisa) at 121°C, 15 lb_2_/in^2^ for 15 min [43].

### 2.4. BCP Immersed in SBF and SIF

The analysis of the interaction between BCP and the simulated fluids was performed by placing a 1/10 ratio (weight/volume) between BCP and the fluids (SBF or SIF) at 37°C for 12 h. After this time, the solid was filtered and washed with 50 mL of deionized water and subsequently dried at 65°C for 12 h in a forced convection oven (Memmert) [43]. The sample was sieved and placed in a crucible to be subjected to 400°C for 1 h in a muffle furnace (Felisa) to promote BCP dehydration to analyze its stability after its interaction with the fluids [43, 44].

### 2.5. Determination of Purity by the Ash Method

In a clean crucible, the weight is measured and subsequently placed in a muffle (Felisa) at 350°C for 30 min to eliminate moisture. After the crucible has cooled to room temperature, it is placed in a desiccator, and this process is repeated until the weight of the crucible is constant. Then, 0.3 g of the sample is placed and calcined in a muffle at 600°C for 30 min to remove organic matter. The amount of inorganic matter (%IM) present in the sample is determined using the following expression [45, 46]:

(1) $$\begin{array}{c} \%\text{ IM}=\frac{M_{0}-M_{f}}{M_{0}}*100, \end{array}$$

where *M*_0_ and *M*_f_ are the initial and final mass of the sample (g).

### 2.6. Characterization of BCP

Attenuated total reflectance–Fourier-transform infrared spectroscopy (ATR-FTIR) was used to analyze the biosorbent before and after adsorption. The infrared spectrum analyzed the wave number ranging from 4000 to 400 cm^−1^ using a Thermo Scientific Nicolet iS10 analyzer with a total of 32 scans which were achieved with a resolution of 4 cm^−1^. In addition, X-ray diffraction patterns (XRD) were obtained in a diffractometer (Ultima IV Rigaku) sing monochromatic Cu Kα radiation (*λ* = 0.1541 nm) operating at 40 kV and 30 mA. The diffractograms were registered in the 2*θ* range from 4° to 80° in 2*θ*, using a step of 0.03°. The scanning electron microscopy images (SEM) and the energy-dispersive X-ray pectroscopy (EDS) (SEM-EDS-EDX) were obtained in a JOEL spectrometer (6510 pus).

## 3. Results

### 3.1. Purity Determination by the Ash Method

The determination of %IM present in the biomaterial synthesized and analyzed after immersion in different gastric fluids is shown in Table 1. It was observed that biomaterial had an increase in the presence of %IM when it was in contact with SBF and SIF, due to the transformation or union of the organic matter contained in the biomaterial [18, 26, 39, 42]. As can be seen, the %IM values obtained were high, since the ash method focuses on determining the concentration of inorganic matter, making it a practical and effective option to determine the purity in terms of crystallinity of the biomaterial being low (∼12%) [7, 47]. This result indicated that the biomaterial is composed mainly of inorganic matter in the form of calcium phosphate compounds. Through the XRD technique, it will be possible to identify the type of apatites obtained. In the case of the increase in %IM of the biomaterial after its immersion in SBF and SIF, we could infer that it is due to two situations: (1) the presence of a greater quantity of Ca^2+^ and POPO_4_^−^ ions in the SBF solution compared to SIF and (2) the temperature to which they were exposed after immersion allows a better interaction between these ions and the organic matter of the biomaterial to increase the amount of %IM [7, 39, 42, 47].

### 3.2. ATR-FTIR of Biomaterials

**Figure 1:**
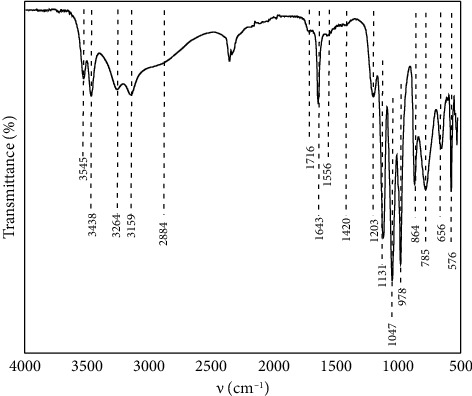
ATR-FTIR spectra of BCP.

To know the functional groups present on the surface of BCP, they were investigated by ATR-FTIR spectrum, as shown in Figure 1, and bands could be observed at 3545 and 3264 cm^−1^ indicating asymmetric and symmetric stretching vibrations of water molecules [8, 12, 16, 29]. In addition, stretching vibrations of water molecules from hydroxyl groups correspond to 3438 and 3150 cm^−1^ [12, 16, 24]. The band at 2880 cm^−1^ is assigned to H-O-H bending of water molecules [19, 29]. The band at 1716 cm^−1^ is associated to HPO_4_^2−^, and the band at 1639 cm^−1^ is attributed to the bending vibrations of the O-H bond in the water molecules present in the structure of the biomaterial [8, 12, 13, 24, 29]. The band corresponding to the bending mode of H_2_O was observed at 1556 cm^−1^, and the band at 1420 cm^−1^ indicates the presence of the HPO_4_^2−^ group through the O-H bending mode [4, 7, 16, 24, 29]. Besides, the bands at 1203 and 864 cm^−1^ correspond to the stretching and bending mode of P-O-H, respectively [4, 8, 12, 16, 24, 29]. The adsorption bands at 1131, 1047, and 978 cm^−1^ correspond to the specific peak of the PO_4_^3−^ group and the presence of P_2_O_7_^4−^ [8, 12, 16, 24, 29]. The band at 785 cm^−1^ is attributed to the release of water and the presence of P_2_O_7_^4−^ and the P-O deformation mode of the phosphate group were observed at 656, 576, and 531 cm^−1^ [7, 12, 13, 16, 19, 29]. As observed in the BCP spectrum, there are the characteristic adsorption bands of the synthesized apatite, nevertheless, due to the presence of a large amount of inorganic matter in the biomaterial, the presence of other apatites similar to the one synthesized may be present.

### 3.3. BCP Immersed in Physiological Fluid

**Figure 2:**
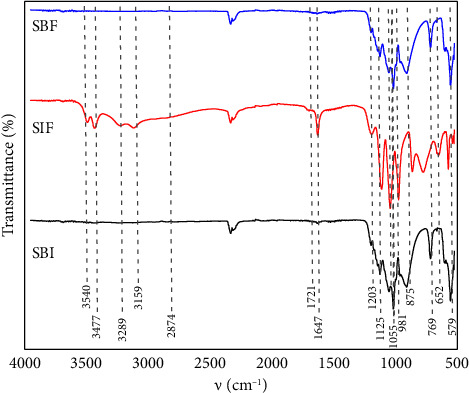
BCP immersed in SBF, SIF, and combination of fluids (SBI).

Figure 2 shows the ATR-FTIR spectra of BCP after immersion in different physiological fluids. It can be identified in the spectrum of BCP immersed in SBF, and the bands at 3545, 1716, 1420, 1203, 864, and 531 cm^−1^ indicate that there is a change in the functional groups on the apatite surface such as OH and HPO_4_^3−^, mainly. In addition, weak bands were present with changes in wavenumbers at 3470, 3272, 3155, 164,3 and 1516 cm^−1^ [7, 18, 26, 27]. The bands at 1135 and 1062 cm^−1^ correspond to stretching vibrations associated with the P-O group that are associated with the transformation of BCP to DCPA [7, 8, 18, 19]. Besides, a weak band appears at 1132 cm^−1^ which is assigned to ν_6_′_6_´y ν_6_^″^ of the HPO_4_^2−^^−^ group which is due to the transformation of BCP to HAP [7, 8, 18]. The weak bands at 1156 and 973 cm^−1^ correspond to P-OH stretching and PO stretching, respectively. These bands are due to the transformation of BCP to DCPA and HAP [7, 8, 18]. The decrease of the band to 913 cm^−1^ is attributed to the P-OH stretching group (ν_3_) of HPO_4_^2−^ that belongs to BCP [19, 26, 27]. The band at 785 cm^−1^ is attributed to the release of water, and the band at 665 cm^−1^ is assigned to the PO bending [7, 8]. P-O deformation mode of the phosphate group was observed in the bands 595 and 553 cm^−1^ [7, 8, 19, 26, 27]. In this interaction between BCP and SBF after heating, changes in the intensity and position of the adsorption bands were observed, indicating a transformation toward the formation of HAP, ACP, or the increase in the proportion of DCPA in the biomaterial which indicates that the amount of inorganic matter increased after the interaction [7, 8, 44, 48].

However, for BCP immersed in SIF (Figure 2), it is observed that the changes in intensity and adsorption bands are smaller compared to BCP. This is due to the change in position of the functional groups in the biomaterial and the disappearance of the band assigned to the HPO_4_^2−^ group. The bands at 3540 and 3286 cm^−1^ indicate asymmetric and symmetric stretching vibrations of water molecules, respectively [7, 8, 27]. In addition, stretching vibrations of water molecules from hydroxyl groups at 3477 and 3159 cm^−1^ observed that these vibrations correspond to BCP [7, 8, 27]. A shoulder was observed at 2874 cm^−1^ and is assigned to H-O-H bending of water molecules [19, 29]. The band corresponds to the bending mode of H_2_O and bending (O-H) in the HPO_4_^2−^ group at 1716, 1556, and 1420 cm^−1^, respectively [16, 24, 29]. The band at 1647 cm^−1^ is attributed to the bending vibrations of the O-H bond in the water molecules present in the structure of the sample [8, 19]. Besides, the bands at 1203 and 875 cm^−1^ indicate stretching and bending mode of P-O-H, respectively [4, 8, 19, 26]. The adsorption bands at 1125, 1055, and 991 cm^−1^ represent the specific peak of the PO_4_^3−^ group [4, 7, 8, 18, 19, 26]. The band at 769 cm^−1^ is attributed to the release of water, and P-O deformation mode of the phosphate group was observed at 652, 579, and 536 cm^−1^ [4, 7, 8, 19, 26, 27]. This indicates that the changes occur on the surface of the BCP due to the interaction with SIF and subjected to 400°C; they presented a slight transformation into DCPA, octacalcium phosphate (OCP) or HAP, unlike the BCP immersed in SBF unlike the BCP before immersion in the fluids, in addition to considering pancreatin participation in the apatite transformation process [26, 44].

Figure 2 shows the ATR-FTIR spectrum of BCP after immersion in SBF, SIF, and combination of fluid (SBI) observing the drastic changes in the BCP surface in the adsorption bands at 3600 cm-1 to 1700 cm-1, 1420, 864, and 785 cm-1 indicating the transformation of BCP into others apatite (ACP or HAP) [7, 8, 18, 26, 27]. The other changes were presented in the band at 1638 cm^−1^ and are attributed to the bending vibrations of the O-H bond in the water molecules present in the structure of the biomaterial [8, 19]. Besides, the band at 1215 cm^−1^ was also observed corresponding to the stretching of P-O-H [8, 26]. The adsorption bands at 1130, 1056 cm^−1^, and together with the shoulder at 992 cm^−1^ are attributed to the specific peak of the PO_4_^3−^ group [8, 18, 19, 26, 27]. The P-O deformation mode of the phosphate group was observed at 612 (shoulder), 559, and 520 cm^−1^ [7, 8, 19, 26, 27]. In addition, bands appeared at 1162, 1025, and 820 cm^−1^ that are assigned to the HPO_4_^2−^^−^ group, ν_3_ (PO_4_) stretching, and P-O stretching, respectively [8, 18, 26]. The transformation of BCP with the mixture of physiological fluids together with the temperature of 400°C shows that the biomaterial is almost entirely transformed into inorganic matter, which indicates that there is a synergy effect compared to the interaction of SBF and SIF separately; in addition, apatite could have bioactivity and could dissolve in the gastrointestinal tract to be used in medical applications [4, 9, 20, 37, 41, 44].

### 3.4. XRD of BCP After the Interaction With Physiological Fluids

**Figure 3:**
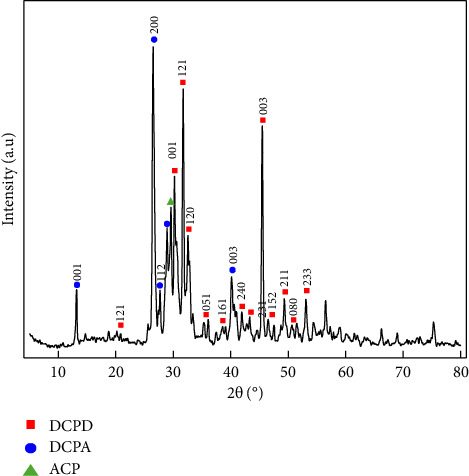
XRD pattern of BCP.

In Figure 3, the BCP diffractograms can be observed with intense peaks approximately at 13.2°, 20.7°, 26.5°, 27.6°, 29.7°, 30.4°, 32.0°, 32.7°, 36.0°, 38.7°, 41.9°, 41.0°, 43.4°, 45.4°, 47.2°, 49.3°, 51.0°, and 53.2°. The peaks that appear at 26.5°, 27.6°, and 32.7° are characteristic of DCPD (JCDPS 11-293) and DCPA (JCPDF 70-360) [4, 19, 30, 32, 48–50]. The other peaks present in the pattern are characteristic of BCP indicating that the synthesized sample has the presence of different apatites, being predominantly composed of BCP [12, 13, 16–18, 24, 28, 29, 48, 49]. Additionally, the peaks with lower intensity at 20.7°, 43.4°, 45.4°, and 47.2° were associated with Ca_2_P_2_O_7_ (JCPDF 00-009-0345), probably due to the transformation of HPO_4_ to calcium pyrophosphate [51, 52]. It was also found that the peaks at 29.7°, 36°, 38.7°, 43.3°, and 47.2° correspond to the presence of CaCO_3_ in the biomaterial [51, 52].

**Figure 4:**
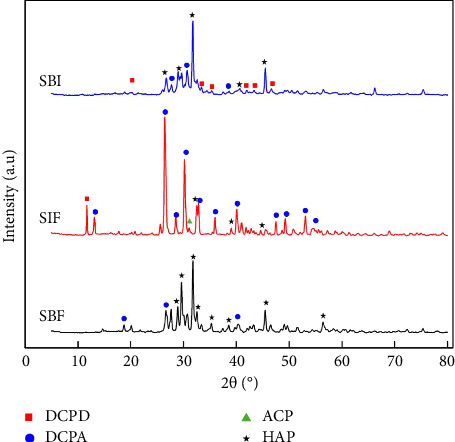
XRD diffraction pattern of the BCP after immersion with different simulated fluids.

Figure 4 shows the diffractograms of BCP after it was immersed in the different physiological fluids. It can be observed in the BCP pattern in SBF peaks at 18.7°, 26.6°, 28.9°, 29.6°, 31.8°, 32.6°, 35.3°, 38.6°, 40.4°, 45.5°, and 56.4°. The interaction between apatite and the fluid caused changes in the structure because the peaks at 29.6°, 31.8°, and 45.5° were observed which are the characteristic of HAP (JCPDS 9-432), implying that part of BCP is transformed into HAP due to the stimulation of precipitation [48]. This transformation is significant, since HAP is a key component of natural bone, and the transformation capacity is relevant for its potential applications in bone repair and bioactivity and this allows an increase in the amount of inorganic matter present in the biomaterial [4, 7, 18, 20, 26, 27, 50, 53]. The interaction of BCP with SBF was found that the peaks at 18.7°, 29.6° and 45.5° correspond to the transformation of HPO_4_ to Ca_2_P_2_O_7_, although the intensity of these peaks is lower compared to those that are characteristic of the apatites present in the biomaterial [51, 52].

In the case of the interaction between BCP and SIF, the XRD pattern shown in Figure 4 exhibits peaks at 11.7°, 13.2°, 26.4°, 28.5°, 30.2°, 32.6°, 32.9°, 36.0°, 39.0°, 40.1°, 45.5°, 47.5°, 49.3°, 53.1°, and 54.7°. Significant changes in intensity were observed along with the disappearance of some peaks in the diffractogram; however, the amount of inorganic matter has a slight increase compared to BCP. This interaction caused BCP to transform into DCPA (the spikes 13.2°, 30.2°, 26.6°, 32.9°, 36°, and 40.1°) due to the change in pH due to the presence of SIF [7, 18, 19, 26, 52, 54]. In addition, there is also a transformation from BCP to HAP because peaks are observed at 28.5°, 32.9°, and 53.1° [19, 51, 52]. In the case of the interaction of BCP with SIF, peaks were found at 32.6°, 32.9°, 36.39°, 45.5°, and 47.7° corresponding to the formation of Ca_2_P_2_O_7_ from HPO_4_ [51, 52]. Finally, the XRD patterns of BCP with the interaction with SBF and SIF showed peaks at 20.2°, 26.7°, 27.7°, 28.9°, 29.7°, 30.8°, 32.0°, 32.6°, 33.5°, 35.3°, 38.6°, 40.4°, 42.5°, 44.3°, 45.4°, 46.5°, 49.6°, and 51.5° which are characteristic peaks of HAP, DCPA, and DCPD indicating that certain portions of the sample were transformed into different amorphous calcium phosphates [7, 19, 52, 54]. The presence of Ca_2_P_2_O_7_ and CaCO_3_ was detected with XRD due to the lack of high intensity of the peaks of both compounds, and no characteristic bands of these functional groups were observed in the biomaterials with ATR-FTIR.

### 3.5. SEM of BCP After the Interaction With Physiological Fluids

**Figure 5:**
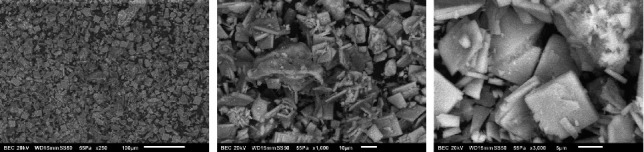
BCP micrographs.

In Figure 5, the micrograph of the BCP surface shows a combination of different morphologies with different shapes and sizes of the plate and elongated crystals; although pointed shapes are also present, different amorphous shapes similar to those shown in the HAP can also be seen, which is confirmed by the XRD analysis, where characteristic peaks of apatites were presented [4, 9, 12, 16, 19, 24, 27, 29, 36].

**Figure 6:**
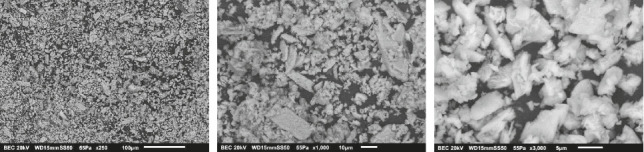
Micrograph of the BCP surface after immersion in SBF.

**Figure 7:**
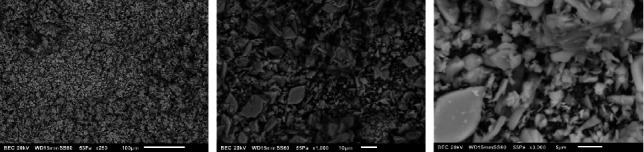
Micrograph of BCP after the interaction with SIF.

Figure 6 shows the micrograph of the BCP surface after the interaction with SBF observing more square shapes with agglomerates, leaving their original pointed and elongated morphology that are representative of only BCP; therefore, it can be said that in this case, a transformation occurs toward DCPA due to the new square morphology characteristic of this apatite and in HAP due to the amount of agglomerates on the surface, indicating that there is a transformation due to the presence of SBF as analyzed in the XRD patterns [4, 7–9, 17, 26, 27]. The BCP surface after interaction with SIF is shown in Figure 7. It could be noticed that it has a morphology with agglomerates and a more square morphology, indicating that BCP did not remain stable. Although elongated and pointed morphologies can be observed, the square morphology and agglomerates are representative of DCPA, implying that the biomaterial interaction can be transformed into DCPA when immersed in SBFs or electrolytic solutions under certain conditions, such as SIF with pancreatin, which was used in this part of the experiment [4, 8, 9, 17, 18, 26, 27].

**Figure 8:**
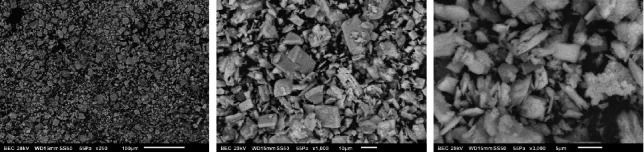
Micrograph of BCP after the interaction with SBF and SIF.

When BCP was immersed in both SBF and SBF (Figure 8), a fairly defined morphology similar to square agglomerates could be observed due to the interaction with both physiological fluids; it can be transformed into DCPA because one of the key factors that can affect the stability of a calcium phosphate is the pH. A change in this was observed during immersion in the fluids from 5.2 to 7 at the time of interaction [4, 7–9, 17, 21, 27, 55].

### 3.6. EDS of BCP After the Interaction With Physiological Fluids

Table 2 shows the EDS results obtained from the sample average of the different biomaterials after their interaction with physiological fluids; it was observed that for BCP before immersion, it was mainly composed of C, O, Na, Ca, and P, with its Ca/P ratio being 1.32. This value indicates that the synthesized BCP contains amorphous calcium phosphates, implying that the biomaterial is not completely pure, since pure brushite should have a Ca/P value equal to 1.0 [3, 5, 7, 10, 16, 17, 22]. It is also mentioned that these values can be associated with the transformation of BCP into other calcium phosphate phases, such as HAP and ACP [2, 4, 8–10, 14, 18, 32]. It is also mentioned that these values can be associated with the transformation of BCP into other calcium phosphate phases, such as HAP and ACP [9, 10, 14, 20].

In the case of the analysis carried out on BCP after the immersion in SBF, an increase of almost double the presence of C and Na was observed; nevertheless, there is a decrease of more than 50% in the presence of O and the presence of Cl in the biomaterial, indicating that the interaction of BCP with SBF undergoes changes in its structure. The Ca/P ratio was 1.46, which is the characteristic of calcium-deficient HAP [9, 10, 18, 20]. Therefore, BCP when immersed in SBF was partially transformed into PAH, as revealed in the XRD analysis.

When the biomaterial is immersed in SIF, the presence of C and O is practically the same as that of synthesized BCP, with the presence of Na decreasing by 50%, in addition to the presence of K in the biomaterial. The ratio was determined to be 1.12. This value indicates that the apatite is DCPA, as revealed in XRD characteristic peaks. The peaks of DCPA were determined as BCP, which, being acidic calcium phosphates, could be transformed into more stable calcium phosphates when placed in *in vivo* media with a pH of around 7 [3, 5, 10, 22, 28, 56, 57].

Finally, in Table 2, the interaction between SBF and SIF with BCP showed that there is a slight increase in the presence of C along with a loss of 26.8% in the biomaterial, in addition to the presence of both Cl and K along with a 50% increase in Na. On the other hand, the Ca/P ratio was 1.38, which is similar to that of BCP without any interaction with physiological fluids, indicating the presence of amorphous phosphates. This consistent with the XRD results, where the characteristic peaks of DCPD, DCPA, ACP, and HAP were analyzed [2, 4, 8–10, 14, 18, 22, 32].

## 4. Conclusion

In the present work, the synthesis of other BCP apatites were achieved by the coprecipitation method with a purity comparable with other synthesis methods having an amount of inorganic matter greater than 85%. BCP presented a light color which was maintained when immersed in SIF, while when interacting with SBF, there was a change to dark gray, and finally in the combined immersion sample, the color was light gray. Furthermore, the amount of inorganic matter in BCP was determined after its interaction with physiological fluids, showing an increase of 9.25% (SBF), 3.27% (SIF), and 11.75% (SIB), indicating changes in the BCP structure. In the characterization of the biomaterial by ATR-FTIR, changes in the functional groups on the biomaterial surface were observed. After immersion in the different fluids, the greatest change in SBI was observed due to a change in pH and the addition of elements such as K and Cl. In the interaction with SBF and SIF, there are changes in the position of the bands of these functional groups, indicating the presence of different calcium phosphates. Peaks of DCPD, DCPA, ACP, and HAP were observed in the diffractograms of the biomaterials, corroborating the structured changes. The characteristic Ca/P ratio of each of the calcium phosphates present in the biomaterial was determined in the EDS results. Pointed crystals were also observed in BCP, which transformed into amorphous, rectangular flat crystals and agglomerates upon immersion in the fluids. Based on these results, it can be mentioned that BCP is not pure and is not stable for use in regeneration; however, it was discovered that it can be transformed into other calcium cements that can be used in regenerative medicine.

## Figures and Tables

**Table 1:** Percentages of IM present in biomaterials in different simulated fluids [42, 43].

Sample	IM (%)
BCP	88.6
SBF	96.8
SIF	91.4
SBI	99.0

**Table 2:** EDS of BCP after immersion in simulated fluids.

% wt.						
Sample	C	O	Na	K	Cl	Ca/P
BCP	10.01	53.59	0.84	—	—	1.32
SBF	24.65	34.74	1.63	—	1.31	1.46
SIF	9.67	53.05	0.41	2.79	—	1.12
SBI	11.52	39.22	1.70	1.63	0.92	1.38

## Data Availability

The data that support the findings of this study are available on request from the corresponding author. The data are not publicly available due to privacy or ethical restrictions.
